# Non-cytotoxic Dityrosine Photocrosslinked Polymeric Materials With Targeted Elastic Moduli

**DOI:** 10.3389/fchem.2020.00173

**Published:** 2020-03-13

**Authors:** Christopher P. Camp, Ingrid L. Peterson, David S. Knoff, Lauren G. Melcher, Connor J. Maxwell, Audrey T. Cohen, Anne M. Wertheimer, Minkyu Kim

**Affiliations:** ^1^Department of Biomedical Engineering, University of Arizona, Tucson, AZ, United States; ^2^Applied Biosciences GIDP, University of Arizona, Tucson, AZ, United States; ^3^BIO5 Institute, University of Arizona, Tucson, AZ, United States; ^4^Department of Materials Science & Engineering, University of Arizona, Tucson, AZ, United States

**Keywords:** photocrosslinking, tyrosine, artificial protein, hydrogel, elastic modulus, non-cytotoxic

## Abstract

Controlling mechanical properties of polymeric biomaterials, including the elastic modulus, is critical to direct cell behavior, such as proliferation and differentiation. Dityrosine photocrosslinking is an attractive and simple method to prepare materials that exhibit a wide range of elastic moduli by rapidly crosslinking tyrosyl-containing polymers. However, high concentrations of commonly used oxidative crosslinking reagents, such as ruthenium-based photoinitiators and persulfates, present cytotoxicity concerns. We found the elastic moduli of materials prepared by crosslinking an artificial protein with tightly controlled tyrosine molarity can be modulated up to 40 kPa by adjusting photoinitiator and persulfate concentrations. Formulations with various concentrations of the crosslinking reagents were able to target a similar material elastic modulus, but excess unreacted persulfate resulted in cytotoxic materials. Therefore, we identified a systematic method to prepare non-cytotoxic photocrosslinked polymeric materials with targeted elastic moduli for potential biomaterials applications in diverse fields, including tissue engineering and 3D bioprinting.

## Introduction

Polymeric biomaterials that are designed to mimic the mechanical properties of tissue matrices can direct cellular behaviors, such as proliferation and differentiation (Engler et al., 2006; Guvendiren and Burdick, 2012; Chaudhuri et al., 2016). Polymers can be chemically crosslinked to form polymer-network materials, such as hydrogels, that mimic the elastic moduli of natural tissues, ranging from 1 kPa in brain tissue to over 100 kPa in bone (Engler et al., 2006; Chaudhuri et al., 2016). Furthermore, crosslinking strategies that rapidly form hydrogels on the order of seconds to minutes are advantageous to precisely fix complex material shapes. However, rapid chemical crosslinking strategies often result in clusters of densely and sparsely crosslinked regions because polymers quickly crosslink before the reagents are well-mixed, resulting in diminished mechanical properties (Kroll and Croll, 2015; Gu et al., 2017, 2020). Therefore, a rapid crosslinking strategy with improved crosslinking homogeneity is necessary to fabricate tissue-mimicking polymeric biomaterials.

Photochemical crosslinking is a potential method to improve the consistency of crosslinking density in rapidly formed polymeric materials because solutions can be thoroughly mixed prior to activating photocrosslinking reagents. Dityrosine photocrosslinking is an attractive and simple approach that exploits light and photoinitiator-activated phenolic coupling of tyrosyl groups within synthetic or natural polymers (Aeschbach et al., 1976; Fancy and Kodadek, 1999; Partlow et al., 2016). The photoinitiator tris(2,2′-bipyridyl)ruthenium(II) ([Ru(II)bpy_3_]^2+^) and persulfate oxidizing agents are often used for rapid dityrosine photocrosslinking (Elvin et al., 2005, 2010; Fang and Li, 2012; Ding et al., 2013; Jeon et al., 2015; Yang et al., 2015; Kim et al., 2017; Zhang et al., 2017; Min et al., 2018; Sakai et al., 2018; Khanmohammadi et al., 2019; Lim et al., 2019). [Ru(II)bpy_3_]^2+^ functions by absorbing visible light and reducing a persulfate anion to reach a higher energy state, [Ru(II)bpy_3_]^3+^. The persulfate anion is consumed in the reaction through decomposition from S_2_${\text{O}}_{8}^{2-}$ to ${\text{SO}}_{4}^{2-}$ and ${\text{SO}}_{4}^{•-}$. [Ru(II)bpy_3_]^3+^ oxidizes tyrosyl phenyl groups into free radicals that spontaneously dimerize (Nickel et al., 1994). The complete process of [Ru(II)bpy_3_]^2+^ (Ru)-mediated crosslinking occurs on the order of seconds to minutes, and the strategy has been used to form polymeric materials, such as hydrogels, with elastic moduli ranging from 6 kPa to over 100 kPa (Elvin et al., 2010; Ding et al., 2013; Zhang et al., 2017). The range of potential elastic moduli makes the technology sufficient to form biomaterials that mimic particular tissue matrices.

Despite the benefits of Ru-mediated dityrosine photocrosslinking, there is disagreement about the possible cytotoxicity of the Ru and persulfate crosslinking reagents (Annabi et al., 2017). In preparation of dityrosine photocrosslinked polymeric materials, persulfate concentrations have ranged from at least 1 to 200 mM, and Ru concentrations from 0.1 to 3 mM (Elvin et al., 2005, 2010; Fang and Li, 2012; Ding et al., 2013; Jeon et al., 2015; Kim et al., 2017; Zhang et al., 2017; Min et al., 2018; Sakai et al., 2018; Khanmohammadi et al., 2019; Lim et al., 2019). Persulfates are strong oxidizing agents that can stress cell membranes and lead to an increased rate of apoptosis (Song et al., 2017). Ru is an intercalator that can affect DNA and cell replication (Ang and Dyson, 2006; Gill et al., 2016). Due to the potential cytotoxicity of Ru and persulfates, dityrosine photoinitiators including riboflavin or flavin mononucleotide (Kato et al., 1994; Applegate et al., 2016; Donnelly et al., 2017; Liu et al., 2018), and Rose Bengal (Spikes et al., 1999) have been used as alternatives. However, these approaches come at the cost of slower crosslinking that limit potential time-sensitive biomaterials applications, such as stereolithographic 3D bioprinting, where rapid crosslinking is beneficial (Melchels et al., 2010; Bajaj et al., 2014; Valot et al., 2019). Therefore, an evaluation of Ru photocrosslinking parameters is necessary to carefully utilize Ru crosslinking technology and guide the rapid production of non-cytotoxic dityrosine photocrosslinked polymeric materials.

We hypothesized that elastic moduli of Ru-mediated photocrosslinked materials can be targeted by controlling concentrations of Ru and persulfate where the limiting reagent dictates the elastic modulus. Moreover, we expected that hydrogels prepared from formulations where both reagents are at limiting concentrations could enhance the survivability and growth of cells compared to excessive reagent concentrations. To investigate the Ru-mediated fabrication of non-cytotoxic dityrosine polymeric materials with targeted elastic moduli, we used artificial proteins as model polymers. Advantages of artificial polypeptides include precise genetic engineering for well-controlled Tyr molarity in the system and monodispersed biosynthesis to reduce batch-to-batch variations of polymer lengths (Kim et al., 2015; Yang et al., 2017; Dzuricky et al., 2018). We utilized elastin-like polypeptides (ELP) incorporated with tyrosine residues, ELP(Tyr), as an unstructured polymer model (Roberts et al., 2015) to form dityrosine photocrosslinked hydrogels.

**Figure 1:**
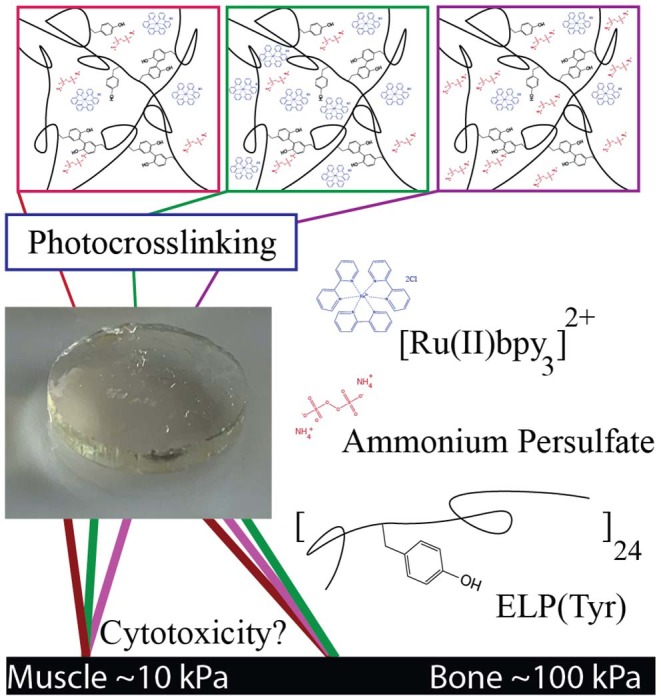
Schematic of tyrosine-photocrosslinked hydrogels with targeted elastic moduli. Multiple formulations with various concentrations of Ru and persulfate can potentially produce hydrogels with similar elastic moduli. Each formulation may have a different degree of cytotoxicity depending on crosslinking reagent concentrations. Reagents in images are an artistic representation of higher concentrations than what is optimal for target G′ and are not representative of actual molar concentrations.

Artificial protein ELPs are typically composed of repeating (GX_aa_GVP) pentapeptide sequences, where X_aa_ can be any amino acid except proline (Urry et al., 1985). Tyrosine and alanine residues comprise the X_aa_ positions, [(GAGVP)_2_-GYGVP-(GAGVP)_2_]_24_, to construct ELP(Tyr). Tyrosine residues allow for photocrosslinking, and together with alanine residues, set the lower critical solution temperature (LCST) at 29°C to utilize the inverse transition cycling (ITC) method, a purification strategy that exploits the reversible, temperature-dependent, phase separation property of ELP (Meyer and Chilkoti, 1999; Christensen et al., 2013). The biocompatibility of ELP-based scaffolds, micelles, and hydrogels has led to its utilization in biomedical applications (Urry et al., 1991; Simnick et al., 2007), making ELP(Tyr) a suitable polymer to examine potential cytotoxic effects of Ru and persulfate concentrations when used to form dityrosine photocrosslinked materials with targeted elastic moduli (Figure 1).

In this study, we controlled the elastic modulus (G′) of photocrosslinked hydrogels by tuning Ru and ammonium persulfate (APS) concentrations with a constant ELP(Tyr) concentration to control the tyrosine molarity in each crosslinking formulation. Hydrogels with similar G′ that were formed by different crosslinking formulations were evaluated in cytotoxicity assays to understand changes in cytotoxicity due to differences in Ru and APS concentrations. The cytotoxicity assay tests the utility of Ru-mediated dityrosine crosslinked hydrogels in applications for which buffer exchange of the hydrogel is difficult, such as *in-situ* hydrogel applications (Bang et al., 2018) where crosslinking reagents necessarily come into direct contact with cells. This study demonstrates a systematic approach to prepare non-cytotoxic biomaterials with targeted elastic moduli given any appropriate tyrosyl-containing polymer by controlling the concentrations of Ru and APS used during photocrosslinking.

## Materials and Methods

### ELP(Tyr) Synthesis

The ELP(Tyr) plasmid (Figure S1) was kindly provided by the Dr. Harvinder Gill laboratory (Ingrole et al., 2014). The pET-24 a(+) ELP(Tyr) plasmid was transformed into BL21(DE3) competent *Escherichia coli* (*E. coli*) cells (New England Biolabs, Ipswich, MA). One colony was amplified in 10 mL LB media with 50 mg/L kanamycin overnight at 37°C and 220 rpm. The overnight culture was centrifuged at 3,000 × g for 15 min at 4°C. The pellet was resuspended in 2 mL LB media, and 500 μL of cell culture was added to 1 L terrific broth media with 50 mg/L kanamycin. Cultures were incubated at 37°C and 220 rpm for 24 h, centrifuged at 6,000 × g for 5 min to harvest cells, then frozen at −80°C for at least 1 h. Cell pellets were resuspended in pH 7.5 phosphate buffer and lysed using a Branson Sonifier 250 (Branson Ultrasonics, Danbury, CT). Cell debris were removed by centrifuging at 25°C, 10,800 × g for 15 min. ELP(Tyr) was purified using the inverse transition cycling method (Meyer and Chilkoti, 1999) using the described protocol (Ingrole et al., 2014). The cell lysate was heated to 40°C then centrifuged at 40°C, 8,000 × g for 15 min to pellet phase-separated ELP(Tyr). The pellet, including ELP(Tyr), was resuspended in 4°C deionized water to dissolve ELP(Tyr). The resuspended pellet solution was then centrifuged at 4°C, 20,000 × g for 15 min to remove impurities. The supernatant was transferred to a new bottle and the pellet was discarded. The process was repeated for two more cycles and purity was confirmed via SDS PAGE (Figure S2). The purified protein was dialyzed in 4.5 L deionized water and changed every 3+ h 7 times at 4°C. Then the soluble fraction was centrifuged and lyophilized, yielding about 350 mg ELP(Tyr) per 1 L cell culture. Lyophilized ELP(Tyr) was stored at −20°C.

### Photocrosslinked ELP(Tyr) Hydrogels

The photocrosslinking solution was prepared containing 10% w/v ELP(Tyr) in pH 7.5 phosphate buffer and was mixed with various concentrations of ammonium persulfate and tris(2,2′-bipyridyl)ruthenium(II) chloride hexahydrate (Sigma-Aldrich, St. Louis, MO, USA). Molds were printed by a Formlabs FORM 2 printer with Dental SG resin (Formlabs, MA, USA). Cylindrical 14 mm diameter × 2 mm height molds were prepared for rheology. Cylindrical 20 mm diameter × 1 mm height and 8 mm diameter × 1 mm height molds were prepared for cytotoxicity assays. The crosslinking solution filled the molds and was then irradiated at a distance of 10 inches under a 24 W, 460 nm, 14 × 14 LED array for 10 min. The hydrogels were removed from the molds and stored in glass scintillation vials covered with aluminum foil.

### Rheology

The viscoelastic mechanical properties of the hydrogels were analyzed by small amplitude oscillatory shear rheology on a Discovery Hybrid Rheometer 2 (TA Instruments, USA) with a sandblasted 8 mm parallel plate geometry and a sandblasted stage. Inertia, friction, and rotational mapping calibrations were performed prior to each experiment. A Peltier temperature-controlled stage maintained 4°C for all rheology testing. The cylindrical hydrogel was cut to 8 mm diameter, transferred to the stage, and aligned with the geometry before lowering the gap height until the axial force reached 0.05 N. Strain sweeps were performed from 0.01 to 1,000% shear strain at a constant 10 rad/s angular frequency. G′ was determined by averaging the data points within the linear viscoelastic region of the strain sweep (Figure S3). Frequency sweeps were performed from 0.01 to 100 rad/s at a constant 0.1% shear strain where it showed the chemical gel behavior, G′ > G″. The statistical data analysis was conducted using Prism 8 software (GraphPad Software Inc., CA, USA; Tables S1–S3).

### Fibroblast Culturing

For the hydrogel cytotoxicity and the MTT (3-(4,5-dimethylthiazol-2-yl)-2,5-diphenyltetrazolium bromide) assay, low passage (per ATCC guidelines) human neonatal foreskin fibroblast cells (ATCC CRL-2097) were cultured in Iscove's Modified Dulbecco's Media (IMDM) with 10% fetal bovine serum (FBS) without antibiotics at passage 12. Culturing conditions were kept at 37°C, 5% CO_2_, and over 95% humidity. Assay plates (either 96 well or 12 well tissue culture treated plates) were seeded from fresh cultures that were harvested at about 75% confluence, as follows: 12-well tissue culture plates were seeded at 125,000 cells/1 mL, and 96-well tissue culture plates at 10,000 cells/100 μL per well.

### Hydrogel Cytotoxicity Assay

A live dead viability/cytotoxicity kit (Invitrogen Cat# L3224) was used to assess hydrogel cytotoxicity. Prepared hydrogels remained protected from light at 4°C for up to 16 h before cytotoxicity testing. Prior to introduction to the 70–80% confluent monolayer, each hydrogel was soaked in 70% ethanol for 10 min protected from light at room temperature, soaked in 3 mL culture media for 5–10 min twice, then gently placed into a 12 well dish. Bright field images were taken at 48 h of co-culture with human neonatal foreskin fibroblast cells (ATCC CRL-2097), then hydrogels were gently removed from the 12 well plates. Culture media was carefully discarded without disturbing the monolayer. Cells were washed three times using 1 mL of dye solution (4 μM Ethidium homodimer-1 and 0.4 μM Calcein AM dye from the kit in D-PBS). After the wash, the dye solution was removed and discarded. A final application of 500 μL dye solution was added and the plate was incubated at room temperature for 1 h, then wrapped in foil and protected from light until imaging on the Bio-Rad ZOE Fluorescent Imager.

### MTT Assay

Tissue culture treated 96 well plates were seeded with 10,000 fibroblast cells/100 μL of media (IMDM + 10% FBS). After 48 h, the plate was treated with varying final concentrations of freshly prepared sterile filtered solutions as follows: ammonium persulfate (APS) (0.75 mM, 0.25 mM); [Ru(II)bpy_3_]^2+^ (Ru) (125, 9 μM); as well as combinations: (9 μM Ru + 0.25 mM APS, 9 μM Ru + 0.75 mM APS). Each treatment was conducted in duplicate and placed in the incubator for 24 h. After 24 h, all media was removed and replaced with 100 μL phenol red free IMDM + 10% FBS. Cells were exposed to 1.2 mM MTT solution (Invitrogen Cat# M6494) and incubated for 4 h at 37°C. Per the manufacturer's rapid protocol, after incubation, 75 μL of media was removed and 50 μL of standard cell culture dimethyl sulfoxide (DMSO) (Invitrogen Cat# D12345) was added to each well, gently mixed and incubated at 37°C for 10 min. After 10 min, the plate was placed in a standard plate reader, shaken for 20 s and read at 540 nm. The assay was repeated on 4 separate days.

## Results and Discussion

To investigate targeted hydrogel elastic moduli (G′), photocrosslinked ELP(Tyr) hydrogels were prepared using assorted molar concentrations of Ru ([Ru]) and APS ([APS]), then evaluated using rheology. The cytotoxicity of hydrogels from different photocrosslinking formulations with similar G′ were examined to develop a method to prepare non-cytotoxic materials using Ru-catalyzed dityrosine photocrosslinking.

### Targeted Hydrogel Elastic Moduli With Multiple Material Formulations

**Figure 2:**
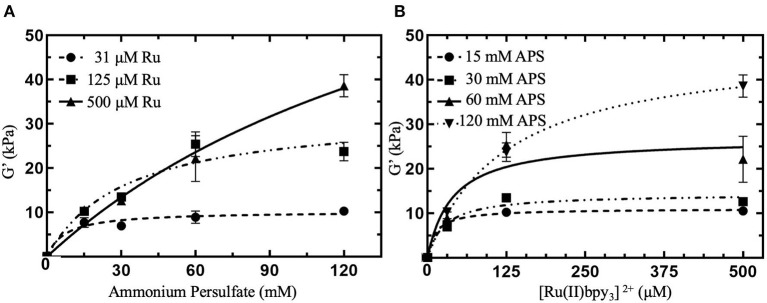
Shear elastic modulus (G′) of photocrosslinked polymeric hydrogels with various concentrations of crosslinking reagents. G′ were collected from linear viscoelastic region from small amplitude oscillatory shear measurements (Figure S3) and averaged (*N* = 3 for each data point). **(A)** G′ for hydrogels with APS molar concentrations, [APS], ranging from 15 to 120 mM with constant [Ru] between 31 and 500 μM. **(B)** G′ for hydrogels with [Ru] varied from 31 to 500 μM with constant [APS] ranging from 15 to 120 mM. All hydrogels contained 10 w/v % ELP(Tyr) dissolved in pH 7.5 phosphate buffer and were subject to 10 min of blue light photoactivation for crosslinking. Error bars represent standard deviation. Curves are least square fitting to the hyperbolic model that assumes the independent variable is a reagent that reaches saturating concentrations. **(B)** Is the reformatting of data in **(A)** with the x-axis relevent to [Ru] to investigate constant [APS]. For formulations with 0 mM [Ru] or [APS], we assumed G′ = 0 kPa since the hydrogel was not formed.

Typical concentrations of Ru and APS used in dityrosine photocrosslinked hydrogels range from 100 to 1,000 μM Ru and 10 to 100 mM APS (Elvin et al., 2010; Fang and Li, 2012; Ding et al., 2013; Zhang et al., 2017; Min et al., 2018). The conversion of [Ru(II)bpy_3_]^2+^ to [Ru(II)bpy_3_]^3+^ consumes APS, thus we expect G′ can be controlled by modulating [APS] in the crosslinking formulation. We investigated variable [APS] from 15 to 120 mM with [Ru] held constant at 125 μM (squares in Figure 2A). We observed that a particular G′ can be targeted below 24 kPa by modulating [APS] up to 60 mM. There was no significiant difference between G′ with 60 mM APS and 120 mM APS (*P* = 0.769, Table S2), indicating that either a limited molarity of tyrosine or Ru was keeping G′ from increasing between 60 and 120 mM APS. To determine whether G′ plateaued due to limited [Ru] or [tyrosine] in the crosslinking formulation, we prepared hydrogels with higher concentrations of Ru, the same concentration of ELP(Tyr), and the same range of APS concentrations.

Photocrosslinked hydrogels were prepared with a 4-fold higher [Ru] to explore a possible increase in hydrogel G′. When [Ru] was increased from 125 to 500 μM at 120 mM APS, G′ of hydrogels were enhanced from ~24 to 38.6 ± 2.5 kPa (triangles in Figure 2A). Contrasting with 125 μM Ru, G′ (22.2 ± 5.2 kPa) for 500 μM Ru was enhanced by 74% when increasing APS from 60 to 120 mM. We attempted to examine the maximum G′ for 500 μM Ru by increasing [APS] beyond 120 mM, but these hydrogels fractured during crosslinking, making further hydrogel measurements not reproducible (Figure S4). Consequently, for ELP(Tyr) hydrogels with 500 μM Ru, [APS] can be modulated to target a particular G′ up to 40 kPa.

To investigate further control of hydrogel G′ using Ru, we tested a 4-fold decrease in [Ru] from 125 to 31 μM. G′ of prepared hydrogels with 31 μM Ru ranged from ~8–10 kPa (circles in Figure 2A), and showed no significant differences in G′ for [APS] between 15 and 120 mM (*P* > 0.05 for all comparisons, Table S2). In summary, 31 μM Ru caused G′ to plateau at all measured APS concentrations (circles in Figure 2A), 125 μM Ru caused G′ to increase between 15 and 60 mM APS but plateau between 60 and 120 mM APS (squares in Figure 2A), while for 500 μM Ru, G′ increased continuously without plateauing (triangles in Figure 2A). This confirms that [Ru] must be high enough (≥500 μM) for APS to be used to modulate G′ up to 40 kPa.

Furthermore, we investigated controlling G′ by holding [APS] constant and modulating [Ru] from 31 to 500 μM to ascertain whether both Ru and APS can act as limiting reagents (Figure 2B). In crosslinking formulations with 15 mM APS, all hydrogels had similar G′ ranging from ~8 to 11 kPa (circles in Figure 2B; *P* > 0.05 for all comparisons, Table S3). For constant 30 and 60 mM APS, there was an increase in G′ when [Ru] was increased from 31 to 125 μM (*P* = 0.0022; *P* < 0.0001, Table S3). However, G′ plateaued when [Ru] was increased from 125 to 500 μM (*P* > 0.05, Table S3). While 120 mM APS was held constant in crosslinking formulations, G′ continously increased from 10.3 ± 0.8 kPa at 31 μM Ru to 38.6 ± 2.5 kPa at 500 μM Ru (inverted triangles in Figure 2B; *P* < 0.0001, Table S3). Altogether, G′ was enhanced when [Ru] increased between 125 and 500 μM for constant 120 mM APS but plateaued when [APS] was 60 mM or below. Thus, controlling the concentration of Ru can be used to target G′ of ELP(Tyr) hydrogels when the APS concentration is not limiting.

When either [Ru] or [APS] is held constant, the other reagent concentration can be modulated to change hydrogel G′. While it has been shown that G′ of Ru-mediated dityrosine crosslinked materials can be altered by modulating persulfate or polymer concentrations in crosslinking formulations with high [Ru] (≥1 mM) (Jeon et al., 2015; Yang et al., 2015), with these results, it can be concluded that [Ru], [APS], and the tyrosyl-incorporated polymer concentration can all control G′ of Ru-mediated dityrosine photocrosslinked hydrogels independent of an excess concentration of other parameters.

Multiple photocrosslinking formulations with different concentrations of Ru and APS can target similar G′ of polymeric materials because Ru and APS can each function as a limiting reagent with respect to G′. For example, 31 μM Ru limits G′ for APS between 15 and 120 mM, resulting in ~8–10 kPa hydrogels (circles in Figure 2A), while 15 mM APS limits G′ for Ru between 31 and 500 μM, also resulting in ~8–11 kPa hydrogels (circles in Figure 2B). Additionally, optimal formulations can be prepared that contain limiting concentrations of both reagents that target a specific G′, such as 8–11 kPa (Figure S5). Although multiple formulations can be used to prepare polymeric materials with similar G′, excess reagents can impact hydrogel cytotoxicity. Therefore, it is necessary to investigate how reagent concentrations that prepare hydrogels can adversely affect cytotoxicity to inform if they are suitable to prepare biomaterials.

### Cytotoxicity Analysis of Hydrogels Prepared by Multiple Crosslinking Formulations

To examine potential cytotoxic effects of different formulations when preparing Ru-mediated crosslinked polymeric hydrogels with similar elastic moduli (G′), we prepared three formulations of various [Ru] and [APS] that each target G′ to ~10 kPa (Figure 2): low [Ru] and low [APS] (31 μM Ru, 15 mM APS), high [Ru] and low [APS] (125 μM Ru, 15 mM APS), and low [Ru] and high [APS] (31 μM Ru, 120 mM APS).

**Figure 3:**
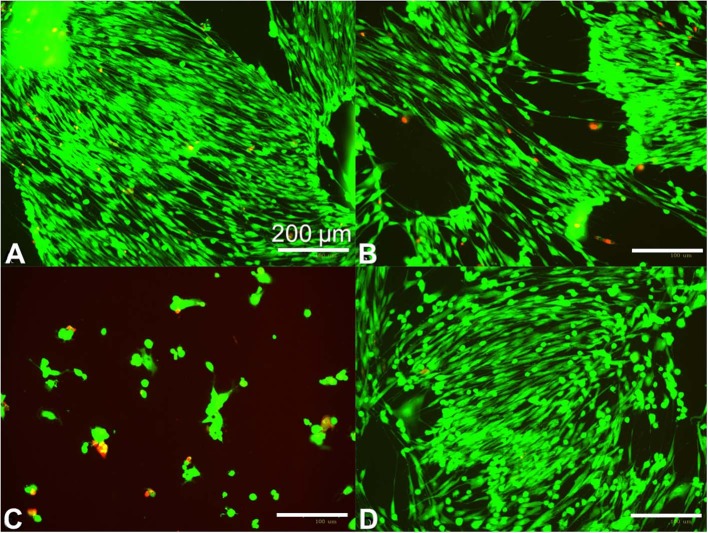
Human fibroblast cytotoxicity/viability assay for ~10 kPa hydrogels prepared using different photocrosslinking formulations. Fibroblasts were seeded at 125,000 cells per well and grown overnight in standard 12 well dishes, then treated at 70–80% confluence with 20 mm diameter × 1 mm height crosslinked hydrogels for 48 h. Hydrogels were prepared with 10 w/v% ELP(Tyr) and 125 μM Ru/15 mM APS **(A)**, 31 μM Ru/15 mM APS **(B)**, and 31 μM Ru/120 mM APS **(C)**. The hydrogel was omitted in the control well **(D)**. Green fluorescence indicates live cells while red indicates dead cells. Bright field images are available in Figure S7.

The prepared hydrogels were applied to human primary fibroblasts at 70–80% confluence in clutures to understand if some formulations can negatively impact cell growth. We found that hydrogels prepared with low APS and either low or high Ru (Figures 3A,B) showed no cytotoxicity compared to controlled cell growth with no hydrogel added (Figure 3D). In contrast, the hydrogels prepared with low Ru and high APS were cytotoxic (Figure 3C). Therefore, the amount of APS leaching out from the hydrogel and coming into contact with the fibroblasts is likely correlated with the increase in cytotoxicity.

We anticipated that the total mass of APS in the hydrogel could be the major factor compared to the APS molar concentration in the hydrogel because the total mass of released APS will increase the APS concentration in a given cell culture volume for the fibroblast cytotoxicity assay. To understand the impact of hydrogel volume on fibroblast cytotoxicity, we compared the 20 mm diameter × 1 mm height hydrogels that caused cytotoxicity (Figure 3C) to 8 mm diameter × 1 mm height hydrogels with various formulations in fibroblast cytotoxicity assays. The hydrogel volume to well volume ratio was 1:3 for the larger hydrogels and 1:20 for the smaller hydrogels. The smaller hydrogels showed no fibroblast cytotoxicity irrespective of formulation after 48 h (Table S4), while the larger hydrogels with 31 μM Ru and 120 mM APS were cytotoxic after 48 h (Figure 3C), indicating that the hydrogel with the greater total mass of APS showed increased cytotoxicity.

To confirm that the mass of APS in hydrogels will impact cytotoxicity, we added the total mass of APS used in the cytotoxic 20 mm diameter hydrogels with 120 mM APS to 8 mm diameter hydrogels, bringing the concentration in the 8 mm hydrogels to 750 mM APS. We found that the hydrogels with 120 mM APS did not disrupt cell growth compared to the control with no hydrogels added (Figures S6A,C), but despite constant G′ and hydrogel volume, hydrogels with 750 mM APS disrupted cell growth (Figure S6B). Thus, the total amount of APS is the major parameter that determines cytotoxicity. Cytotoxicity assays performed by other groups have shown that biomaterials prepared with Ru-mediated crosslinking showed no cytotoxicity (Elvin et al., 2009; Syedain et al., 2009; Lv et al., 2013; Keating et al., 2019). This may be a result of lower amounts of excess APS in crosslinking formulations, testing being conducted on confluent monolayers or cells with low rates of proliferation (Williams et al., 2005), or the testing of hydrogels with smaller volumes and a lower total mass of crosslinking reagents relative to cell media volume to dilute excess reagents.

The direct impact of [Ru] and [APS] on fibroblast cytotoxicity was quantified using MTT assays. We found [Ru] up to 125 μM did not affect fibroblast cytotoxicity, while [APS] even at 0.25 mM showed cytotoxicity (Figure S8). These data indicate that high APS concentrations should be carefully avoided in crosslinking formulations due to acute cytotoxicity. It has been suggested that the consumption of APS in the reaction reduces the toxicity of produced hydrogels compared to the formulation before photoactivation (Elvin et al., 2009). Yet, hydrogels with the higher [APS] depicted in Figure 3C were cytotoxic. In those hydrogels, 120 mM APS is in excess of the concentration necessary to reach ~10 kPa because similar G′ can be obtained with 15 mM APS at the same low Ru concentration (circles in Figure 2A). It is expected that the excess APS was not consumed in the reaction since G′ was limited by low [Ru]. Thus, the hydrogel leaked unreacted APS into the fibroblast media and caused cytotoxicity. The hydrogel cytotoxicity was reduced for smaller hydrogels because the amount of excess APS that leaked out into the media was less than the larger hydrogel prepared with the same [APS] in the constant well volume. However, smaller hydrogels or larger well volumes to reduce [APS] in cell media may not realistically represent possible clinical applications. Therefore, hydrogels that are prepared with just enough APS to target the elastic modulus while avoiding excess [APS] can result in lower unreacted [APS] in the prepared hydrogel for reduced cytotoxicity.

A similar approach to preparing crosslinking formulations is necessary when considering [Ru]. Since Ru is implicated as a DNA intercalator, avoiding excess [Ru] in crosslinking formulations could improve biocompatibility of Ru-mediated photocrosslinked materials. We found that modulation of [Ru] was effective to control G′ such that low [Ru] could consistently target a lower G′ independent of [APS] and excess crosslinking time. Yet, the recyclability of Ru in this reaction has led to speculation that Ru cannot be effectively used to modulate G′ (Syedain et al., 2009). The cyclic conversion of [Ru(II)bpy_3_]^2+^ to [Ru(II)bpy_3_]^3+^ by APS oxidation and back to [Ru(II)bpy_3_]^2+^ by tyrosyl reduction would seem to allow for lower [Ru] to reach the same G′ given more time. However, the activity of Ru was temporally limited for the photocrosslinking of ELP(Tyr), shown by formulations with excess [APS] but lower G′ compared to formulations with the same [APS] but increased [Ru] (circles compared to triangles in Figure 2A). This finding indicates [Ru] and [APS] must be high enough to target a particular G′. Yet, excess [Ru] and [APS] can be avoided for enhanced biocompatibility by systematically evaluating possible formulations to reach a target G′ value for a given tyrosyl-containing polymer.

## Conclusions

We identified a systematic method for utilizing rapid Ru-mediated dityrosine photocrosslinking technology to form non-cytotoxic polymeric materials with elastic moduli (G′) that mimic natural tissues. G′ of hydrogels can be targeted by modulating the concentrations of [Ru(II)bpy_3_]^2+^ (Ru) and ammonium persulfate (APS), potentially due to a temporally limited activity of Ru and the consumption of APS during the reaction. Our results indicate that prototypes of Ru-mediated photocrosslinked polymeric biomaterials can lead to false cytotoxicity results when the ratio of hydrogel volume to cell media volume is small; therefore, cytotoxicity assays should be thorough to investigate volume limitations of biomaterials relative to the surrounding cells given a particular crosslinking formulation. Additionally, monitoring cell growth by beginning the cytotoxicity assay with cells that are not fully confluent instead of 100% confluent monolayers is a better approach for wound healing models and surgical implantation of hydrogels where cell growth around the material is necessary. Finally, Ru and APS should both be set at limiting concentrations to reach a desired G′ such that Ru concentrations are low, and a larger percentage of APS is consumed in the reaction. Taking these steps can unlock the benefits of rapidly preparing Ru-mediated photocrosslinked non-cytotoxic polymeric biomaterials with targeted elastic moduli and temporal control of Ru activation for translation to clinical applications where rapid polymer crosslinking is preferred.

## Data Availability Statement

The raw data supporting the conclusions of this article will be made available by the authors, without undue reservation, to any qualified researcher.

## Author Contributions

MK conceived the project. MK and AW designed the overall experiments. CC, IP, DK, LM, CM, AC, and AW designed and performed the individual experiments. CC, AW, and MK wrote the manuscript. All authors edited the manuscript.

### Conflict of Interest

The authors declare that the research was conducted in the absence of any commercial or financial relationships that could be construed as a potential conflict of interest.
