# Supplementary material for: Non-cytotoxic Dityrosine Photocrosslinked Polymeric Materials With Targeted Elastic Moduli
Source: Front Chem. 2020 Mar 13;8:173. doi: 10.3389/fchem.2020.00173 (PMC7082925; doi:10.3389/fchem.2020.00173)
Supplement: Supplementary file 1 [file Table_1.docx]

**Noncytotoxic dityrosine photocrosslinked polymeric materials with targeted elastic moduli**

Camp, Christopher P.^1^; Peterson, Ingrid L.^2,4^; Knoff, David S.^1^; Melcher, Lauren G.^4^; Maxwell, Connor J.^1^; Cohen, Audrey T.^1^; Wertheimer, Anne M.^2,4,*^; Kim, Minkyu^1,3,4,*^

^1^Department of Biomedical Engineering, University of Arizona, Tucson, Arizona, 85721, USA

^2^Applied Biosciences GIDP, University of Arizona, Tucson, Arizona, 85721, USA

^3^Department of Materials Science & Engineering, University of Arizona, Tucson, Arizona, 85721, USA

^4^BIO5 Institute, University of Arizona, Tucson, Arizona, 85719, USA

Supplementary Material

1. Supplementary Figures and Tables
   1. **Supplementary Figures**
   2. **Supplementary Tables**

**1.1 Supplementary Figures**

**ELP(Tyr):** MGAGVPGAGVPGYGVPGAGVPGAGVPGAGVPGAGVPGYGVPGAGVPGAGVPGAGVPGAGVPGYGVPGAGVPGAGVPGAGVPGAGVPGYGVPGAGVPGAGVPGAGVPGAGVPGYGVPGAGVPGAGVPGAGVPGAGVPGYGVPGAGVPGAGVPGAGVPGAGVPGYGVPGAGVPGAGVPGAGVPGAGVPGYGVPGAGVPGAGVPGAGVPGAGVPGYGVPGAGVPGAGVPGAGVPGAGVPGYGVPGAGVPGAGVPGAGVPGAGVPGYGVPGAGVPGAGVPGAGVPGAGVPGYGVPGAGVPGAGVPGAGVPGAGVPGYGVPGAGVPGAGVPGAGVPGAGVPGYGVPGAGVPGAGVPGAGVPGAGVPGYGVPGAGVPGAGVPGAGVPGAGVPGYGVPGAGVPGAGVPGAGVPGAGVPGYGVPGAGVPGAGVPGAGVPGAGVPGYGVPGAGVPGAGVPGAGVPGAGVPGYGVPGAGVPGAGVPGAGVPGAGVPGYGVPGAGVPGAGVPGAGVPGAGVPGYGVPGAGVPGAGVPGAGVPGAGVPGYGVPGAGVPGAGVPGAGVPGAGVPGYGVPGAGVPGAGVPGAGVPGAGVPGYGVPGAGVPGAGVPGY

**Figure S1.** ELP(Tyr) protein sequence.

**
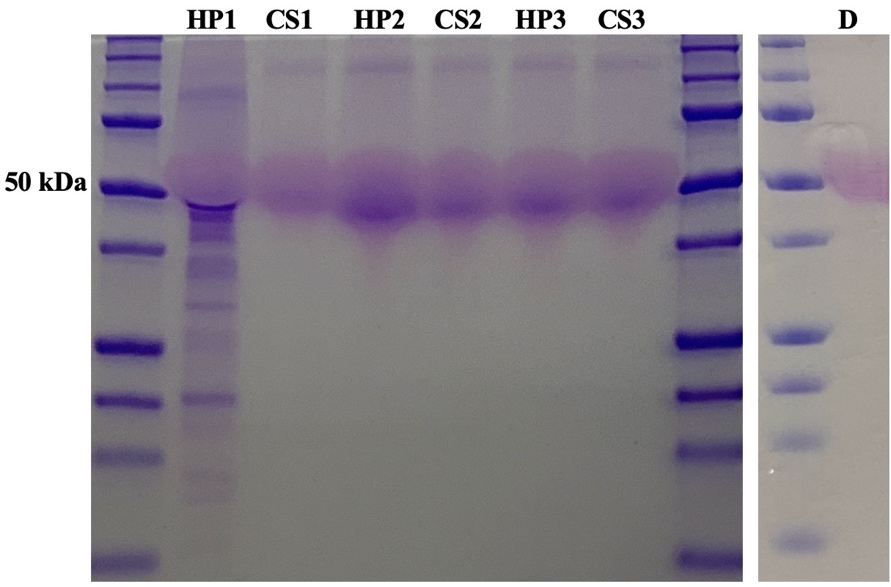
**

**Figure S2.** Purity of ELP(Tyr) analyzed via sodium dodecyl sulfate polyacrylamide gel electrophoresis (12% w/v) after the first purification step of inverse transition cycling purification. **HP1:** First resuspended ELP(Tyr) pellet; **CS1:** Soluble ELP(Tyr) fraction after centrifuging the resuspended first ELP(Tyr) pellet at 4°C; **HP2:** Second resuspended ELP(Tyr) pellet obtained by heating CS1 and centrifuging at 40°C; **CS2:** Soluble ELP(Tyr) fraction after centrifuging the resuspended second ELP(Tyr) pellet at 4°C; **HP3:** Third resuspended ELP(Tyr) pellet obtained by heating CS2 and centrifuging at 40°C; **CS3:** Soluble ELP(Tyr) fraction after centrifuging the resuspended third ELP(Tyr) pellet at 4°C. **D:** Dialyzed ELP(Tyr) solution. CS3 samples were dialyzed against deionized water, 7 times every 3+ hours at 4°C and then centrifuged at 4°C. Soluble ELP(Tyr) fractions were lyophilized and used to prepare ELP(Tyr) hydrogels.

**
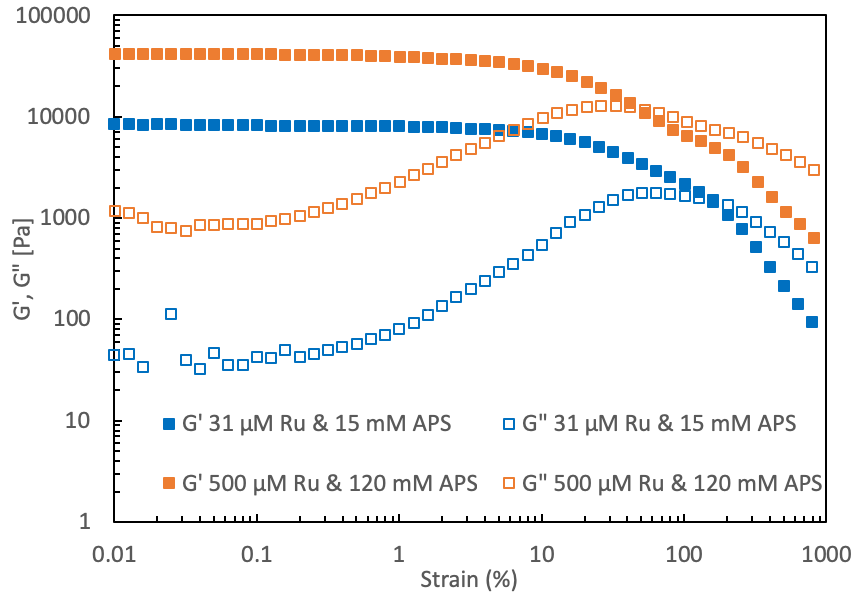
**

**Figure S3.** Strain sweep of hydrogels with 31 μM Ru and 15 mM APS, and 500 μM Ru and 120 mM APS. G’ values between 0.01 rad/s and 0.1 rad/s were averaged to determine G’.

**
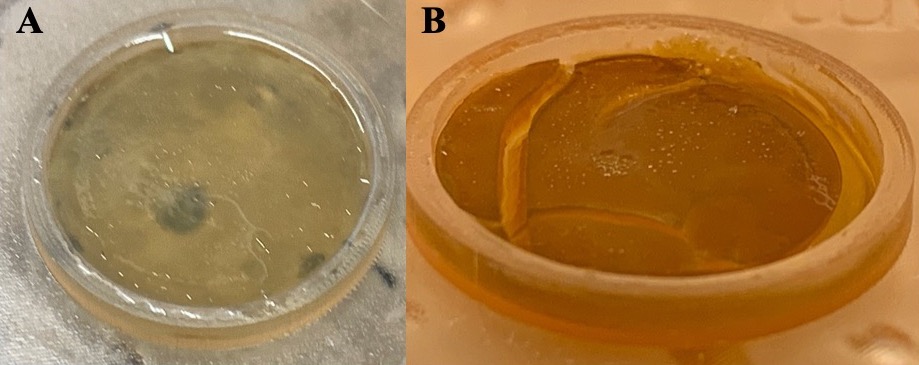
**

**Figure S4.** Hydrogels prepared with 10 w/v% ELP(Tyr) that were used to measure G’ in Figure 2 or test cytotoxicity in Figure 3. **A:** 30 mM APS and 125 μM Ru hydrogel in 3D printed mold after crosslinking and **B:** 240 mM APS and 1 mM Ru fractured in the mold following crosslinking.


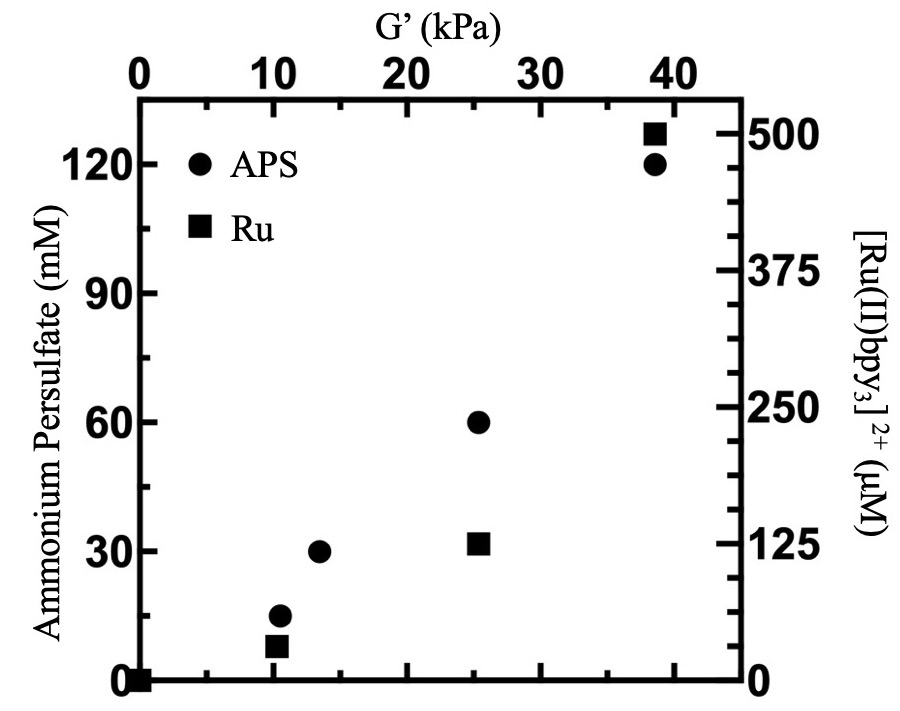


**Figure S5**. Scatter plot of optimized Ru and APS concentrations to reach a target G’. The points for each G’ represent minimal concentrations of each reagent to reach that target G’. For example, to prepare 10 kPa or 25 kPa ELP(Tyr) hydrogels, 31 μM Ru/15 APS and 125 μΜ Ru/60 mM APS are limiting reagents to target that respective G’. For ~15 kPa, 30 mM APS is limiting, and the minimal Ru concentration should be between 31 μM and 125 μM to avoid excess. Note that 500 μM Ru and 120 mM APS were the maximum concentrations tested because of hydrogel fracture during photocrosslinking (see Figure S4), thus one or both reagents may not be limiting at ~40 kPa.


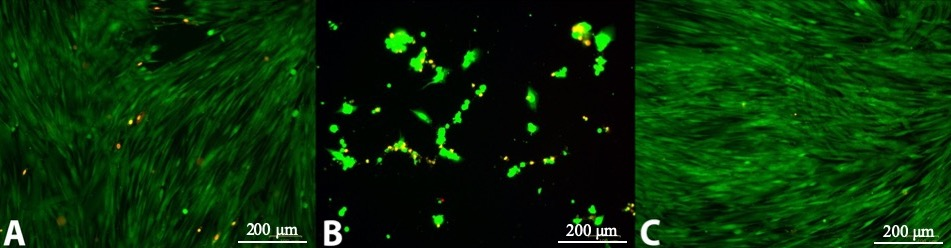


**Figure S6.** Human fibroblast cytotoxicity/viability assay for ~10 kPa hydrogels prepared using different photocrosslinking formulations. Fibroblasts were seeded at 100,000 cells per well in standard 12 well tissue culture dishes, then treated at 70-80% confluence with 8 mm diameter x 1 mm height hydrogels for 48 hours. Hydrogels were prepared with 10 w/v% ELP(Tyr) and **A:** 31 μM Ru/120 mM APS, **B:** 31 μM Ru/750 mM APS, and **C:** Control well without a hydrogel. Green fluorescence indicates live cells while red indicates dead cells.


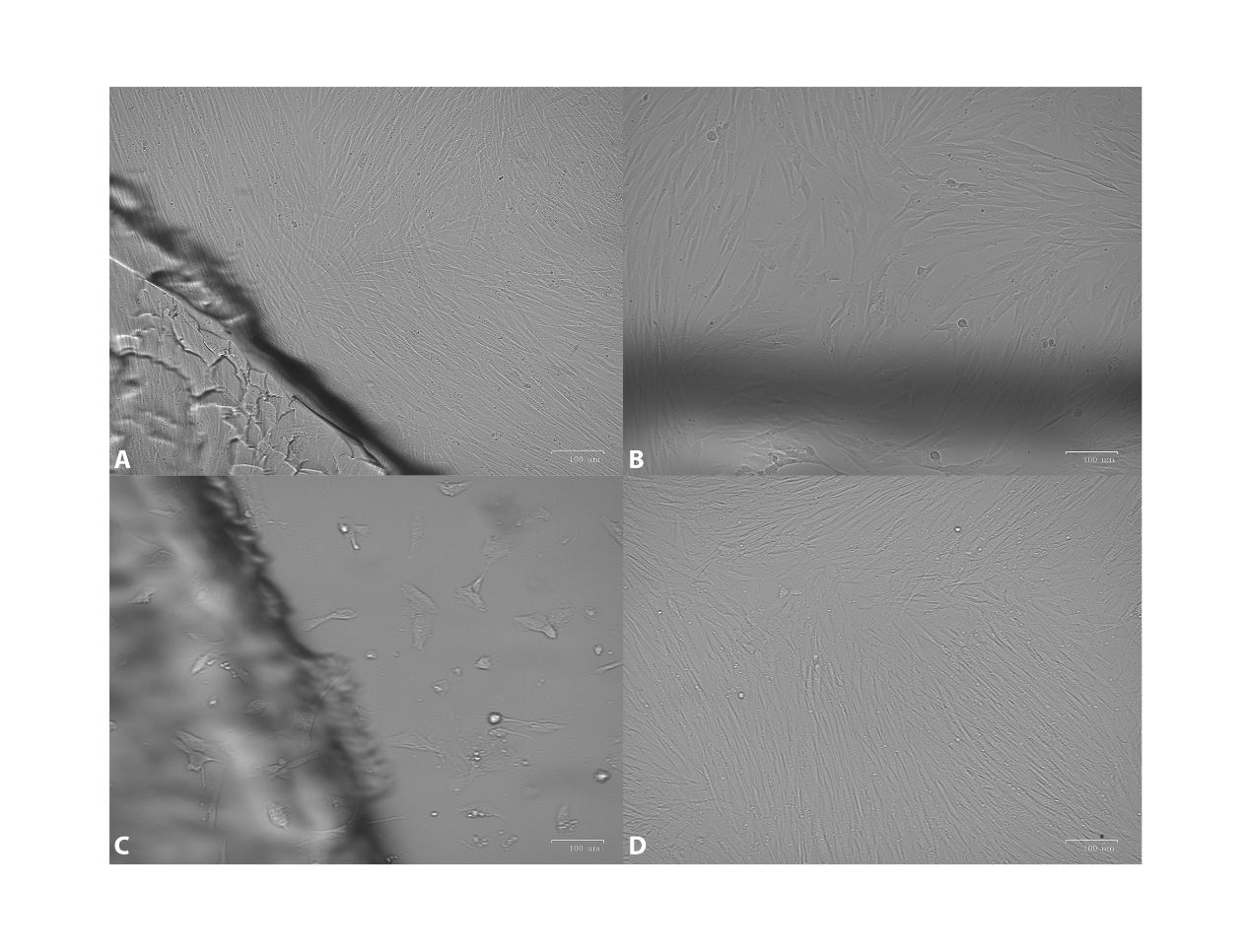


**Figure S7.** Bright field images of human fibroblasts from the LIVE/DEAD assay (Figure 3) at 48 hours. Fibroblasts were seeded at 125,000 cells per well overnight in standard 12 well dishes then treated at 70-80% confluence with 20 mm diameter x 1 mm height crosslinked hydrogels. **A** 125 μM Ru/15 mM APS; **B** 31 μM Ru/15 mM APS; **C** 31 μM Ru/120 mM APS; **D** Control well with no hydrogel.


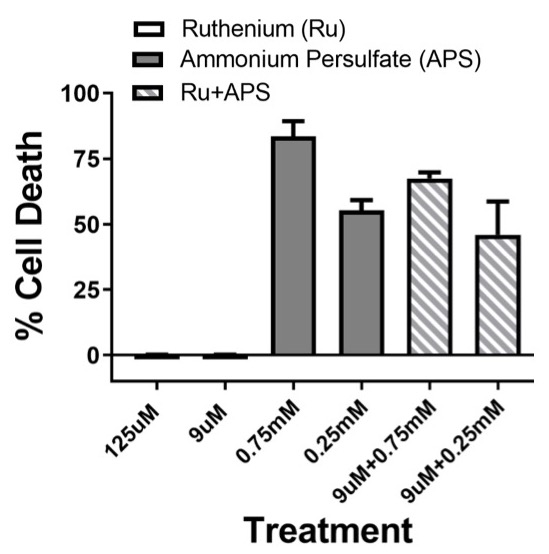


**Figure S8.** Impact of Ruthenium and Ammonium Persulfate Upon Human Neonatal Fibroblasts. Each compound was introduced to cells in duplicate upon confluence, incubated at 37°C with 5% CO_2_ for 24 hours. A standard MTT assay was performed and read at 540 nm. Percent death was calculated based upon untreated cells.

**1.2 Supplementary Tables**

**Table S1.** 2way ANOVA results determined significant variation for different Ru and APS values. α = 0.05.

| Source of Variation | % of total variation | P value | P value summary | Significant? |
| --- | --- | --- | --- | --- |
| APS + Ru | 22.69 | <0.0001 | **** | Yes |
| APS | 40.90 | <0.0001 | **** | Yes |
| Ru | 33.10 | <0.0001 | **** | Yes |

| ANOVA Table | SS | DF | MS | F (DFn, DFd) | P value |
| --- | --- | --- | --- | --- | --- |
| APS + Ru | 712.1 | 6 | 118.7 | F (6, 24) = 27.46 | P<0.0001 |
| APS | 1284 | 3 | 428.0 | F (3, 24) = 99.03 | P<0.0001 |
| Ru | 1039 | 2 | 519.5 | F (2, 24) = 120.2 | P<0.0001 |
| Residual | 103.7 | 24 | 4.322 |  |  |

**Table S2.** Tukey's multiple comparisons test comparing average G’ values for hydrogels with constant [Ru] and variable [APS] for Figure 2A. N=3 for each formulation.

| **Constant Ru** | **Mean Diff.** | **95.00% CI of diff.** | **Significant (P<0.05)** | **Summary** | **Adjusted P Value** |
| --- | --- | --- | --- | --- | --- |
| **31 μM Ru** |  |  |  |  |  |
| 15 mM APS vs. 30 mM APS | 0.7294 | -3.953 to 5.412 | No | ns | 0.9728 |
| 15 mM APS vs. 60 mM APS | -1.226 | -5.908 to 3.457 | No | ns | 0.8873 |
| 15 mM APS vs. 120 mM APS | -2.607 | -7.290 to 2.075 | No | ns | 0.4326 |
| 30 mM APS vs. 60 mM APS | -1.955 | -6.637 to 2.727 | No | ns | 0.6619 |
| 30 mM APS vs. 120 mM APS | -3.337 | -8.019 to 1.346 | No | ns | 0.2286 |
| 60 mM APS vs. 120 mM APS | -1.382 | -6.064 to 3.301 | No | ns | 0.8472 |
|  |  |  |  |  |  |
| **125 μM Ru** |  |  |  |  |  |
| 15 mM APS vs. 30 mM APS | -3.238 | -7.920 to 1.444 | No | ns | 0.2515 |
| 15 mM APS vs. 60 mM APS | -15.15 | -19.83 to -10.47 | Yes | **** | <0.0001 |
| 15 mM APS vs. 120 mM APS | -13.51 | -18.19 to -8.825 | Yes | **** | <0.0001 |
| 30 mM APS vs. 60 mM APS | -11.91 | -16.59 to -7.229 | Yes | **** | <0.0001 |
| 30 mM APS vs. 120 mM APS | -10.27 | -14.95 to -5.587 | Yes | **** | <0.0001 |
| 60 mM APS vs. 120 mM APS | 1.642 | -3.040 to 6.324 | No | ns | 0.769 |
|  |  |  |  |  |  |
| **500 μM Ru** |  |  |  |  |  |
| 15 mM APS vs. 30 mM APS | -2.084 | -6.766 to 2.599 | No | ns | 0.616 |
| 15 mM APS vs. 60 mM APS | -11.66 | -16.35 to -6.981 | Yes | **** | <0.0001 |
| 15 mM APS vs. 120 mM APS | -28.06 | -32.74 to -23.38 | Yes | **** | <0.0001 |
| 30 mM APS vs. 60 mM APS | -9.58 | -14.26 to -4.898 | Yes | **** | <0.0001 |
| 30 mM APS vs. 120 mM APS | -25.98 | -30.66 to -21.29 | Yes | **** | <0.0001 |
| 60 mM APS vs. 120 mM APS | -16.4 | -21.08 to -11.71 | Yes | **** | <0.0001 |

**Table S3.** Tukey's multiple comparisons test comparing average G’ values for hydrogels with constant [APS] and variable [Ru] for Figure 2B. N=3 for each crosslinking formulation.

| **Constant APS** | **Mean Diff.** | **95.00% CI of diff.** | **Significant (P<0.05)** | **Summary** | **Adjusted P Value** |
| --- | --- | --- | --- | --- | --- |
| **15 mM APS** |  |  |  |  |  |
| 31 μM Ru vs. 125 μM Ru | -2.558 | -6.797 to 1.681 | No | ns | 0.3055 |
| 31 μM Ru vs. 500 μM Ru | -2.850 | -7.089 to 1.388 | No | ns | 0.2336 |
| 125 μM Ru vs. 500 μM Ru | -0.2923 | -4.531 to 3.946 | No | ns | 0.9838 |
|  |  |  |  |  |  |
| **30 mM APS** |  |  |  |  |  |
| 31 μM Ru vs. 125 μM Ru | -6.525 | -10.76 to -2.287 | Yes | ** | 0.0022 |
| 31 μM Ru vs. 500 μM Ru | -5.663 | -9.902 to -1.425 | Yes | ** | 0.0075 |
| 125 μM Ru vs. 500 μM Ru | 0.8620 | -3.377 to 5.101 | No | ns | 0.8683 |
|  |  |  |  |  |  |
| **60 mM APS** |  |  |  |  |  |
| 31 μM Ru vs. 125 μM Ru | -16.48 | -20.72 to -12.24 | Yes | **** | <0.0001 |
| 31 μM Ru vs. 500 μM Ru | -13.29 | -17.53 to -9.050 | Yes | **** | <0.0001 |
| 125 μM Ru vs. 500 μM Ru | 3.194 | -1.045 to 7.432 | No | ns | 0.1658 |
|  |  |  |  |  |  |
| **120 mM APS** |  |  |  |  |  |
| 31 μM Ru vs. 125 μM Ru | -13.46 | -17.70 to -9.220 | Yes | **** | <0.0001 |
| 31 μM Ru vs. 500 μM Ru | -28.30 | -32.54 to -24.06 | Yes | **** | <0.0001 |
| 125 μM Ru vs. 500 μM Ru | -14.84 | -19.08 to -10.61 | Yes | **** | <0.0001 |

**Table S4.** Human fibroblast cytotoxicity assay of 8 mm diameter x 1 mm height 10% ELP(Tyr) hydrogels. Various APS and Ru formulations were introduced to 70-80% confluent cells growing in standard 12 well dishes (22.1 mm diameter) with 1 mL media. 100% confluence of monolayer (+++) after 24 hours.

| **Formulation** | **8 mm x 1 mm**  **Hydrogel Viability** |
| --- | --- |
| **125 μM Ru**  **15 mM APS** | +++ |
| **31 μM Ru**  **15 mM APS** | +++ |
| **31 μM Ru**  **120 mM APS** | +++ |
